# A Proposed Planning Concept for Public Open Space Provision in Saudi Arabia: A Study of Three Saudi Cities

**DOI:** 10.3390/ijerph17165970

**Published:** 2020-08-17

**Authors:** Abdullah Addas, Ahmad Maghrabi

**Affiliations:** Landscape Architecture Department, Faculty of Architecture & Planning, King Abdulaziz University, P.O. Box 80210, Jeddah 21589, Saudi Arabia; aamaghrabi@kau.edu.sa

**Keywords:** public open spaces, planning approaches, planning standards, Saudi Arabia

## Abstract

Public open spaces (POS) have an essential positive impact on cities and their residents. These spaces play a critical role in enhancing users’ physical, mental, and social wellbeing. In addition, POS improve city resilience and economic value, and act as part of the city’s visual amenities. The Kingdom of Saudi Arabia is taking many approaches to enhance quality of life in all its cities through initiatives such as increasing the POS area per capita. Several studies have examined the importance of the accessibility of POS in addressing users’ needs. In this study, we measured the per capita area and accessibility of POS in the three megacities Riyadh, Dammam, and Jeddah. We also collected data on user preferences for the use of POS through semi-structured interviews, observations, and an online questionnaire. This study suggests that the country needs to establish its own POS planning approach based on users’ desires and behaviors when using POS, as well as the country’s social characteristics, and to depend not only on standard international planning approaches. The paper recommends considering the possibility of increasing POS by creating typologies of these spaces based on each city’s landscape characteristics. This proposal will have a major impact on city planning and design in Saudi Arabia. In addition, it will make the Saudi cities livable and have a positive impact on the physical, mental, and social wellbeing of the population.

## 1. Introduction

The United Nations included a Sustainable Development Goal (SDG) in the New Urban Agenda in 2016 to create comprehensive, protected, reliable, and sustainable urban settlements [1]. City planning, spatial distribution, ecological amenities, urban context management, the quality of public open spaces (POS), and socio-economic facilities can all enhance urban sustainability depending on how implementation is addressed [2]. Studies have classified POS in the urban context as essential elements to improve the quality of life in cities [3,4,5,6]. Other studies have identified the contribution of POS to achieving social [7,8,9], health [10,11,12], environmental [13,14], and economic benefits [15,16,17]. In addition, in many countries, a POS system has become a necessity because of the advantages for both cities and their residents [18]. Studies have been conducted in several countries, examining people’s needs and perceptions of POS [19]. However, few studies have focused on the Middle East in general, and Saudi Arabia in particular [20]. 

POS has a positive impact on users’ wellbeing, which includes promoting stress reduction, relaxation, and restoration, relying to a large extent upon the provision of open space. Ulrich et al. (1991) report that natural settings restore positive effects and reduce fear, anger, and aggression based on attention restoration theory and stress reduction theory [21]. The urban context of POS and physical activities are what improve mental wellbeing. There are several studies that investigated the relation between the provision of POS and public health. These studies found that the percentage of POS within the urban context has a positive impact with the perceived general health of residents. POS not only offer land uses that offer recreation use to the residents, it has direct and indirect impacts on the users mental, physical and social wellbeing [22,23].

Some studies have examined theories of social justice in city planning and the spatial distribution of POS provisions. These theories allow decision-makers and responsible governmental bodies to adopt planning standards that ensure equal access to POS and spatial justice. 

This study argues that, while the needs and demands of POS could be similar to those described in the Western literature, patterns of usage and planning standards could be different due to the social characteristics and urban context of a country, and to user preferences. This study examines the current POS planning approach in Saudi Arabia. It also highlights how planning standards for Saudi cities could be varied according to social needs and patterns of use of POS, based on users’ points of view, the urban context, and an analysis of the accessibility and per capita area of POS.

This study reviews the literature related to POS spatial planning. It looks at current practice in Saudi Arabia by reviewing the approach of the Ministry of Municipal and Rural Affairs (MoMRA) to POS planning and provision. In addition, the study sheds light on the monetary value of POS provision in the three cities examined, and identifies how much the government would need to spend to meet international planning standards. The empirical analysis conducted, and the data collected, were then reviewed and form the basis of suggestions regarding POS provision, including a conceptual planning approach to evaluate POS provision in Saudi Arabia drawn from analysis of semi-structured interviews, an online survey, and the current level of POS accessibility.

### 1.1. Public Open Space Planning

The urban planning of POS usually takes account of factors such as demographic structure, population and density, social needs, space value, and preferences. It is essential to understand POS urban planning models, as they determine the planning criteria for POS, reflecting the range of benefits that POS can offer to a city and its residents. The size and shape of POS play a critical role in enriching users’ experiences and promoting different activities [24]. A larger open space will provide positive experiences to a wide variety of users [25], including children, adults, and older people [26]. Furthermore, larger open spaces play an important role in cities by fostering biodiversity [27] and mitigating high temperatures [28]. 

Maruani and Amit-Cohen (2007) identified three models of POS planning: the opportunistic model, the space standards model, and the park system model. The opportunistic model for acquisition uses land obtained from donations, demolition, the renewal of recycling sites, and leftover spaces from construction or engineering projects such as road building. This model is not related to planning because it depends on chance [29]. The space standards model is related to the quantitative matching of the size and number of POS to the population and area, and focuses on providing a minimum POS area per inhabitant. This model has been criticized because it neglects social and environmental factors [29]. The third model, the park system model, allows for continuous movement within the system and supports the interrelationship of parks and gardens. It focuses on proximity and user diversity for all types of POS in the city. 

The accessibility of POS is a topic of debate in urban planning because of its relationship with environmental justice and public health [30]. According to Dave (2011), the accessibility of POS is one of the key indicators of improved quality of life in cities. This is because perceptions and needs vary, but it is agreed that access to POS is beneficial for all residents [31]. Accessibility to POS can be defined as “ease with which a resident can reach a given destination” (p. 181) [32]. The concept of accessibility is very broad and depends on the context. In addition, in some cases, it is confused with other related terminology, such as mobility, which refers to the ability to move between places [33]. However, accessibility refers to the way people approach a place [34]. Social sciences consider accessibility to be an attribute of people rather than a transport mode, as well as the integration of services from the users’ viewpoint [35]. Other studies have found that the availability of local parks within walking distance of people’s homes is positively associated with the use of those parks. In contrast, the need to drive to reach a park often deters its use [36,37,38,39,40]. Accessibility to POS can be measured based on proximity, quantity, and quality of urban green spaces [41]. The proximity POS is defined as “a geographic unit’s distance to the closest park, regardless of the park’s size and amenities” (p. 162) [42].

Taking proximity into account when planning neighborhoods is essential for reducing urban sprawl and allowing access to services [43]. Various studies have concluded that proximity or access promote good urban form [44,45]. From an urban economic point of view, POS spatial distribution plays a vital role in maintaining land and property values as well as increasing economic worth [46,47,48].

Proximity of POS was previously used as a measure, on the basis that access to POS a short distance from home or work [49] would encourage positive physical activity. However, other studies identified different measures, such as the number of POS and the total area of parks within 1 km [50]. This study argues that the distance to the nearest POS does not play a significant role in increasing users’ physical activity. According to Sugiyama et al. (2010), the distance to POS is not important. However, the size and activities available are critical [51]. Accordingly, this study argues that the planning of neighborhood POS and urban city-scale parks depends on the users who are attracted to these spaces regardless of physical location, and whether or not the parks are near to residents’ homes. 

According to the World Health Organization (2017), measures of accessibility may include the proximity of POS to users or the community regardless of distance, entry with or without fees, and the point of access, such as gates and paths [52]. When applying objective methods, a usage measure is vital, such as observation of the POS, tracking users, or surveying spaces. Different countries have their own national standards regarding POS provision and accessibility. Natural England recommends that everyone, regardless of their location, has an accessible green space not less than 2 ha in area within a 5-min walking distance (2 km), 100 ha within 5 km, and 500 ha within 10 km [53]. The European Common Indicator of POS does not identify a target but uses a similar metric approach and suggests a 15-min walk. The US Environmental Protection Agency identifies various aspects of green space accessibility, such as POS per inhabitant, the population within a 500 m distance by walkable roads, and the pathways of parks [54]. While the metric of distance is used to measure the accessibility of POS [55] in the United States [56] and Europe [57], this approach does not assess equity in access to POS because such access depends on a range of factors, such as transportation, barriers, and socio-political realities [58]. 

These studies and international planning standards have confined themselves to certain scales of measuring POS accessibility (such as a neighborhood unit or a city) without clarifying the differences between the level of accessibility between a neighborhood and a city-scale park. This limitation leads to restricted integration of accessibility measurements. Western literature has focused on the topic of accessibility to public spaces by proposing different tools for measurement and spatial planning of POS. However, in developing countries, few studies have shed light on this topic, including in Saudi Arabia, which is witnessing growing complexity in the urban environment.

Diversity is a significant measure of the quality of the public realm. It is vital to consider social diversity and geographic location in POS spatial planning [59]. Jacobs argued that considering the urban context where a POS is located is essential, and that having more parks does not always indicate a better quality of life. Diversity can be measured by the activities that a site offers, including recreational elements and the esthetics of the space [60]. Parks, gardens, and playing fields offer different experiences and benefits, and allow users to walk and play, relive memories, escape from their everyday routines, and promote social cohesion [20]. Diversity, according to different studies, offers an experiential choice for users [61]. In urban design approaches, a thriving town has diverse users, spaces, and activities that enhance the residents’ experience and quality of life. Gold (1972) argued that unused parks and gardens in a neighborhood are a significant problem because they adversely affect diversity and heighten the residents’ sense of insecurity [62].

From the review of the literature regarding POS planning models and accessibility, we found that there are different indicators to measure POS distribution. This study applies the basic measurement of POS area per capita. According to de la Barrera, “the most widely used indicator to assess green spaces is their total area in respect to the total population” (p. 212) [25]. Many studies argue that this model is too simplistic because it measures only the relationship between POS area and population numbers. To take the POS spatial distribution into account and gain a realistic point of view of POS distribution in Saudi cities, this study also includes a measure of POS accessibility. The body of literature regarding POS is very rich in Western countries. However, there has been little emphasis on POS accessibility, planning, and design in Saudi cities. This study examines POS accessibility, per capita area, and user preferences. Based on this analysis, the study proposes a new methodology for POS planning in Saudi cities. 

### 1.2. Saudi Arabia Characteristics and Background

This subsection provides background on Saudi Arabia, where this study took place, covering population, immigration, and urban expansion, as well as the Vision 2030 programs that relate to enhancing the urban landscape. It also sheds light on urban planning challenges, social attitudes to POS, leisure, and recreation, and patterns of using POS.

#### 1.2.1. Population

Saudi Arabia is the third-largest Arab country in terms of its land area. The population was 34,218,169 in 2019, including more than 10 million non-Saudi residents. The average annual population growth rate is 2.4% [63]. It is important to highlight that the urban population increased from 48.7% in 1970 to 84.1% in 2019. The non-Saudi population in Saudi Arabia in 2019 was estimated to be about 40% of the total [63], and included workers who had migrated from other countries after the discovery of oil, as well as those seeking better work opportunities, especially with the Vision 2030 programs.

#### 1.2.2. Urban Expansion

Over the last 40 years, the movement of people to cities has resulted in considerable urban expansion and rapid population growth. This growth took place without sufficient preparation in terms of planning regulations or strategies for city planning. In addition, ambitious government social and urban development programs have changed the Saudi lifestyle from being centered on small villages to becoming based on cities. These programs have caused the fragmentation of communities, separation of families, and loss of traditional livelihoods [64]. Social changes also include an increased number of immigrants, especially low-skilled workers of many nationalities [20] who may live in particularly concentrated areas [65]. While the Saudi family structure has come to mirror the nuclear family of industrialized countries to some degree, the number of children in Saudi families is much higher [66]. Thus, Litwak (1965) suggests that the Saudi family should be regarded as a modified, extended family structure [67]. 

These social changes, and the growth in both the resident and migratory populations, coincided with substantial increases in the country’s wealth. The overall effect is that Saudi cities have grown without regulations and effective planning. Jeddah, for example, has grown well beyond the capability of its infrastructure [68,69]. The city currently faces issues with its water supply, sewage system, and roads, with residents overly dependent on cars and large areas with empty, undeveloped land [65]. A shortage of affordable housing means that more than one million people, a third of the population, live in unplanned settlements. These social and planning issues are typical of all large cities in Saudi Arabia. The economy and social fabric of the country cannot continue to develop indefinitely without improvements in the country’s built environment, enabling support for modern economic activities [65].

#### 1.2.3. Vision 2030

With the worsening condition of the social environment, priority was given to residential projects to accommodate the growing population. These projects were characterized by inadequate city planning and management [70]. To address the resulting issues, the government set out Vision 2030, which aims to enhance the urban landscape in Saudi cities through various projects and initiatives. One of these initiatives is the Quality of Life Program, which seeks to create a supportive environment that enhances the lives of all citizens, facilitating their participation in various cultural, recreational, and sporting activities [70].

#### 1.2.4. Urban Planning Challenges

In the last four decades, rapid growth in both the built area and migratory populations has followed the Kingdom’s increase in wealth. As a result, megacities have emerged, with urban sprawl in most of the large cities, including Jeddah, Riyadh, and Dammam. This rapid expansion reflects the limited success of regulatory frameworks and spatial planning that have tried to restrict development to certain parts of cities. 

#### 1.2.5. Leisure and Recreation

Working hours in the country have reduced significantly, and now stand at 40 h per week on average [20]. Friday and Saturday are traditionally the Saudi ‘weekend’. Students have a three- to four-month summer vacation and two weeks for their spring vacation. All employees and government workers have the right to take 30 days of paid vacation each year. Thus, there is great demand and need for recreational and leisure activities. The country recently created the General Entertainment Authority, which is responsible for proposing a range of recreation activities, and 2019 saw the first major recreation programs, such as Jeddah Season and Riyadh Season.

POS form a component of daily recreation and leisure, as residents use these spaces as places for family and friends to gather. In addition, studies that have examined how and why people use public spaces in Saudi cities have revealed similarities with the findings of Western studies, which show that these spaces are used for physical activities and social cohesion [20,71].

#### 1.2.6. Social Attitudes to Public Open Spaces

According to Mandeli (2011), the demands of new markets, together with associated planning processes that were designed to re-shape society, have resulted in a sharp break with the traditional urban environment. It is assumed that the vitality and health of the economy and social transition could not be maintained without changing the shape of the built environment and creating a context that supports a range of modern economic activities [65]. However, gardens are important in Islamic countries, and Muslims are encouraged to create, maintain, and visit gardens, as these enable them to reflect on the afterlife and paradise and are also suitable places for daily prayers. Al-Nassan (2008) explains that the gardens that have been established over many years in Saudi Arabia have several different names, such as Al-Rawdah and Al-Bustan (Orchard), although some gardens have maintained their original names [20].

The Saudi economic system has been integrated with Western economies, together with a way of life modeled on modern Western luxury lifestyles [72]. These changes have transformed society, while shifting integrated communities into dispersed populations through housing and transportation projects. Large-scale development projects, such as large apartment blocks, substantial buildings, and wide streets, were imposed, and new residential areas created [65]. The functional classification of land-use regulations has been enforced by both central and local authorities to ensure physical uniformity and the maximization of economic productivity [59]. Islamic countries often base legislation for the environment on Islamic law, which would include esthetic considerations when constructing new buildings, acoustic privacy, visual privacy, removing public waste, and maintaining public places at a high level of cleanliness, as all these factors can negatively affect the environment [20].

Sidky and Bastawisi (2010) reported on the damage to urban POS caused by poor planning decisions in Egyptian cities, namely rapid expansion in particular harms raised by urban environments and open spaces within and beyond cities [73]. Furthermore, regulations stress the segregation of individuals according to their economic and social status, thereby driving social disintegration [74,75] and producing lifeless neighborhoods [20]. Al-Fahad (2008) found that few people use parks and gardens in Saudi Arabia because of their poor design and maintenance, with the softscape and hardscape failing to meet people’s needs [76]. The result is a lack of sense of community that, in turn, inhibits social interactions between residents and disincentivizes participation in outdoor social and sporting activities [65,77,78].

In Saudi Arabia, the advent of modern lighting has shifted most social activities to the late evening, allowing more people to use outdoor recreational areas [79]. However, the nature of leisure activities here is very different from European and North American norms. These activities are influenced by customs, culture, and traditions. 

The Saudi government acknowledges the importance of outdoor recreation and the need to increase access to POS in Saudi cities, and there are various facilities available for this purpose. However, the demand for recreational facilities far outstrips supply. As people retire in good health, and move into cities from rural areas, more open spaces are needed to reduce congestion and overcrowding. However, meeting this demand is hindered in Saudi Arabia by inadequate design policies, poor planning, and insufficient resources.

#### 1.2.7. Pattern of Using Public Open spaces

Many studies related to POS use present findings in Western and Far-Eastern countries. However, few studies have examined the pattern of using POS in Saudi Arabia [20]. Addas documented the pattern of using POS in Jeddah [20] and other sites in Saudi Arabia [71]. These studies found that type, quality, and design are different in important respects from other locations around the world. The study revealed that users have different perceptions regarding how, when, and why to use POS in Jeddah. Users prefer to travel by private car or taxi to certain destinations in the city instead of using nearby POS. In addition, the study found that users spent a long time in POS and, in some sites, the findings showed that they stayed after midnight, especially on weekends and public holidays.

### 1.3. Public Open Space Provision in Saudi Arabia 

MoMRA is the body responsible for the provision of POS in Saudi cities. It supervises, advises, and controls the municipalities (Amanat) in each city. MoMRA creates regulations and guidelines to support urban design, greening projects, and POS provision. These guidelines help municipalities understand the requirements and standards of POS provision. These standards have been adopted from a range of international standards, and support other related topics associated with enhancing the quality of life in cities, such as city greening (Appendix A (Table A1)).

According to the MoMRA guidelines, in the planning process for a residential subdivision there is a need to provide POS, which can include roads, walkways, plazas, playgrounds, gardens, and parking. The POS should not exceed 33% of the total area. Playgrounds for every 20 residential blocks combined must be a minimum of 400 m^2^ in area. In addition, at the neighborhood scale, there is a need for POS with a minimum of 5000 m^2^ to provide 5 m^2^ per capita for residents. It is essential to highlight that these guidelines do not provide sufficient data about quality or design. In addition, MoMRA does not have a clear strategy of city-scale parks planning and design.

MoMRA has proposed a POS hierarchy for various types of POS with their suggested total area, access method, and catchment distance (Appendix A (Table A2)). This hierarchy was created based on space functions and is applied to all Saudi cities.

POS were neglected in the past and given low priority until the announcement of Saudi Arabia’s Vision 2030. In its key performance indicators, the Vision 2030 National Transformation Program (NTP) highlighted the importance of enhancing the urban landscape in Saudi cities by increasing residents’ access to POS and improving POS availability per inhabitant [71,80]. In addition, the NTP established several programs to support Vision 2030, including the Quality of Life Program, which aims to make Saudi cities more livable [70]. Furthermore, community participation in the decision-making process is very limited, and the NTP aims to increase public participation in the public realm [71,80]. 

## 2. Methodological Approaches

The study methods included case studies, an online survey, and on-site semi-structured interviews which, together, provided the information needed to propose a planning approach for POS in Saudi Arabia. According to landscape architecture research methods, when the aim is to collect data related to users’ preferences in case study, the best tools implement either interviews or questionnaires [81]. The questionnaire design and the semi-structured interviews included the elements required to investigate users’ preferences in relation to POS and to support evidence-based decision-making of the proposed concept. The study also measured POS accessibility and per capita by using geographic information system (GIS) (ESRI, CA, USA) data and observations.

### 2.1. Case Study Approach

**Figure 1:**
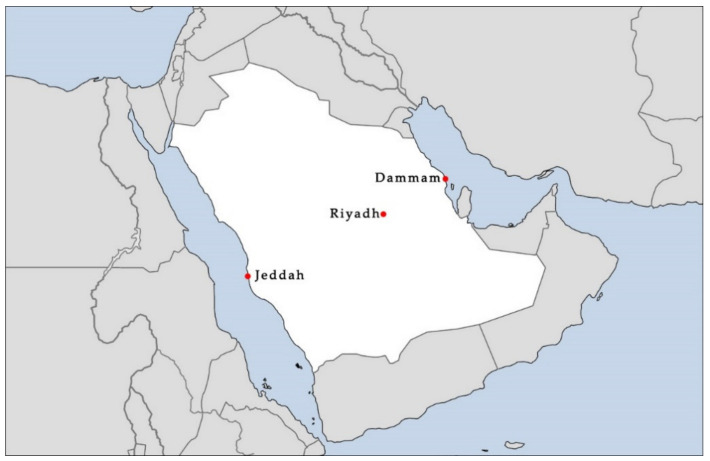
Saudi Arabia map showing each of the study cities’ locations.

To assess the provision of POS in Saudi Arabia, this study examined the physical accessibility of POS in three megacities (Riyadh, Jeddah, and Dammam), as considered in the urban design and planning literature [82,83]. The multiple case study method allowed us to offer a better understanding of the POS provision in Saudi cities [84]. In addition, it helped us develop greater knowledge on the approaches to POS provision developed by MoMRA and the municipalities. These cities were selected because they are the three most substantial cities, and the provision of POS is more advanced here than in other cities. In addition, Jeddah is located on the West Coast, Dammam on the East Coast, and Riyadh in the center of the country (Figure 1). Each of these cities thus has its own unique characteristics. Appendix A (Table A3), shows an overview of each city’s total area, weather, population, and physical environment. The population density in Riyadh, Jeddah, and Dammam is 4659/km^2^, 5400/km^2^, and 2000/km^2^, respectively.

**Figure A1:**
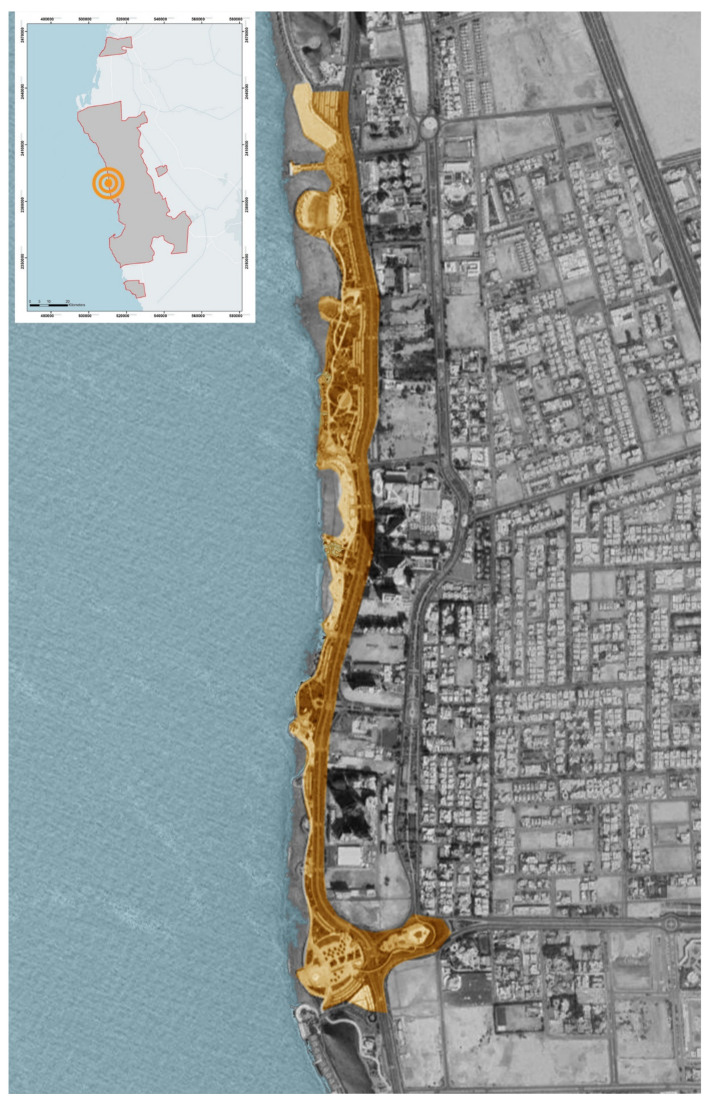
Jeddah study site.

**Figure A2:**
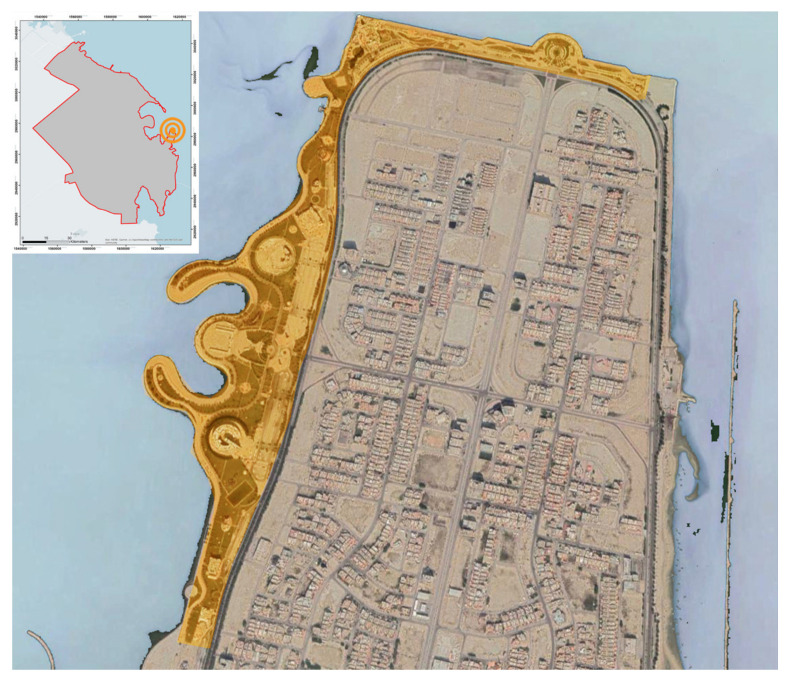
Dammam study site.

**Figure A3:**
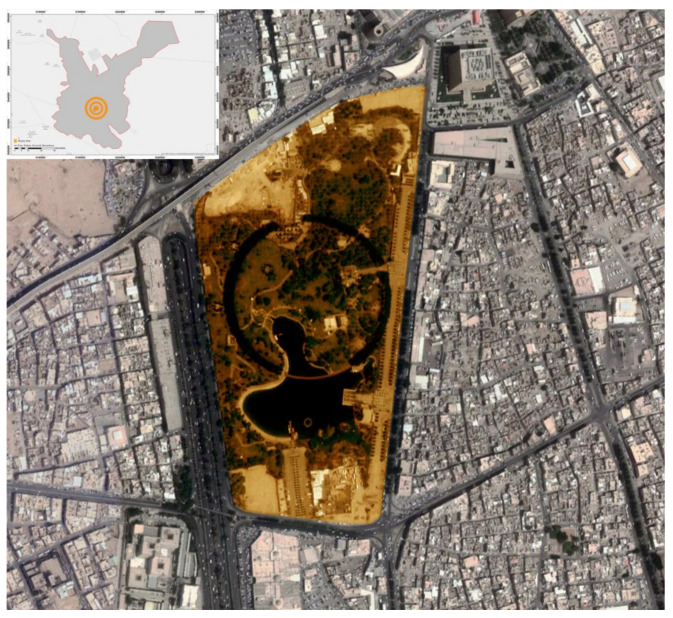
Riyadh study site.

We selected the most common POS destinations for the residents in each city by car survey and observations. The selected spaces were inside the urban growth boundary (UGB) for each city, and the criteria for selection relied on the number of users and the level of attractiveness of each site. In Jeddah and Dammam, the most common destination for residents was the waterfront. We selected the most frequently visited and attractive parts of the waterfronts in Jeddah and Dammam (Appendix A (Figure A1 and Figure A2)), with sport tracks, multiuse areas, public swimming area, vendors and restaurants, and children’s playgrounds. Notably, there are no city-scale parks or gardens that attract residents in these cities, which is the reason why we selected the waterfront. In Riyadh, there were three potential sites for exploration based on the car survey and observations: Al Salam Park, Al Suwaidi Park, and Al Bujairi Park. Al Bujairi Park was excluded because it is part of the Wadi Hanifah project, which involves conservation of the natural environment of the Wadi. Al Salam Park was chosen because it is larger and its level of attractiveness higher than Al Suwaidi Park. This park has a sports track, lake and water sports area, multiuse areas, vendors, and children’s playgrounds (Appendix A (Figure A3)).

### 2.2. On-Site Interviews and Observations 

We conducted seven days of observations at each site, including weekdays and weekends, to fully determine whether the selected sites were used by residents. The observations took place between November 2018 and January 2020. The visual observations of these spaces assisted in understanding the urban environment [85]. A semi-structured interview was also conducted with the users of each site. The questions included the following:

- Where do you live (neighborhood or district)?
- How do you reach this site (transportation method)?
- Do you have a POS near your home? If yes, do you use it?
- Why do you use this POS?
- Why do you use this site (the case study site)?

### 2.3. Online Questionnaire 

The methodology of this study included a web-based questionnaire to investigate resident perceptions and attitudes toward POS. The survey had four main sections: general participant information (city of residence, age, gender, and nationality), pattern of using neighborhood gardens, pattern of using city-scale parks and public recreational facilities, and a measurement of participant preferences for using POS in general (using a Likert scale). The online survey was created using an online survey tool, Google Forms (Google LLC, Mountain View, CA, USA), and ran from March to June 2020. The survey links were posted on our website and different social media pages. 

### 2.4. Accessibility and Per Capita Measurement

This research measured accessibility by using the conventional Euclidean buffer approach, with a simple radius of 300 m or 800 m [33,86,87]. We used this method instead of applying other approaches, such as network analysis, because the study set out to measure accessibility for the whole city without focusing on the level of accessibility in each district. The Euclidean buffer provides an approximate representation of the POS-served areas to support the study’s argument. The 300 m radius agrees with the Accessible Natural Greenspace Standards model. Natural England recommends 300 m as a walking distance to public open spaces, and MoMRA recommends a maximum of 800 m as a buffer zone for POS, which is the transition point from walking to driving based on MoMRA’s standards [80].

This study reviewed the POS inside the UGBs of each city, obtained from the city prosperity index (CPI). All parks and gardens within each city’s urban context were drawn using GIS regardless of their quality of design, area, size, or whether or not they were used. The POS maps for each city relied on satellite images, OpenStreetMap (OpenStreetMap Community, London, UK), Google Earth Pro (Google LLC, Mountain View, CA, USA), and the city strategic master plan were obtained for each city municipality. This study also included car surveys for validation of the location of POS when required to ensure accuracy for the accessibility analysis.

Riyadh population data were obtained from the service guide for Riyadh, which is prepared by the General Authority for Statistics. This report notes that the Riyadh population is 5,236,901, with a 4.1% annual population growth, and 60% of the population are Saudis [88]. Population data for Jeddah were obtained from the Jeddah Urban Observatory JUO (2016) [89]. In 2015, Jeddah had 4,060,591 inhabitants, which equated to 12.8% of the total population of the Saudi Kingdom [80]. The average annual growth rate of the population was 3.8%. Dammam population data were obtained from the General Authority for Statistics. The population is 1,024,409, with 4.1% annual population growth, and 52.7% of the population are Saudi nationals [90].

### 2.5. Data Analysis

**Figure 2:**
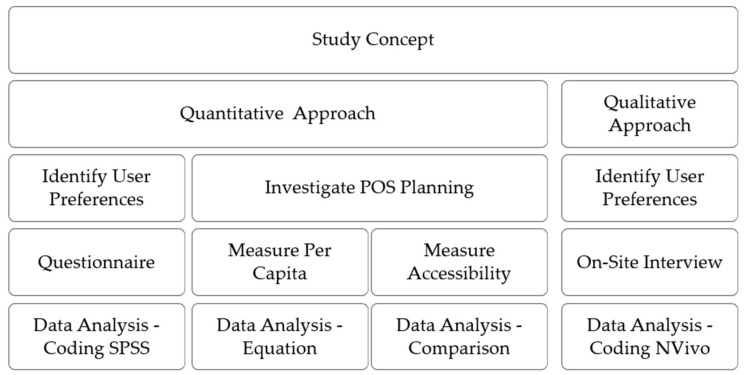
Study methodology process.

This study applied a mixed method, as shown in Figure 2, to understand user preferences regarding POS use and to investigate POS planning in the study cities. This is why qualitative and quantitative data were analyzed separately [81]. The qualitative data was collected using semi-structured on-site interviews to identify user preferences. All the interviews were recorded in Arabic on a voice recorder, and were transcribed and translated by both researchers to ensure that the translation was accurate and did not reflect different meaning. All interviews were coded using the qualitative data analysis software NVivo, Version 11 (Alfasoft, Göteborg, Sweden). Therefore, the authors were aware of what to count as data to help in achieving the study’s aim [91].

The quantitative data collected in this study was used to identify user preferences and to investigate the planning of POS in the study cities. As mentioned, this study used a questionnaire, which was analyzed using SPSS 22.0 (Armonk, New York, NY, USA) to record participant responses. Testing the validity of the study tool (questionnaire) was performed using two methods of validation, namely virtual validity and internal consistency by calculating the correlation coefficients between the score of each of the paragraphs that follow the Likert scale and the total score of these paragraphs. The results showed strong and statistically significant correlation coefficients, thus showing validity and internal consistency. Testing the reliability of the study tool (the questionnaire) using the reliability coefficient returned a Cronbach’s alpha value of 0.507. This value shows the ability of the questionnaire to achieve stable results if distributed to other samples at different times.

Using frequency tables to display the distribution of participants according to personal data (gender, nationality, and place of residence). The total number of study participants who responded and cooperated with the researcher’s request and filled out the questionnaire was 1596 of whom 744 were from Jeddah, 670 from Riyadh, and 182 from Dammam. In addition, using frequencies, arithmetic means, standard deviations, and rank in presenting the participants’ responses to the paragraphs of the second section of the questionnaire. The results were presented separately by city and in total for the three cities combined.

Using a one-way ANOVA test to identify any statistically significant differences in the participants’ responses across the three cities. The results showed the presence of statistically significant differences in the responses of the participants to the questionnaire paragraphs across the three cities.

To evaluate the POS planning in the selected city, the POS area per capita for all cities was calculated according to the following equation:

(1) $$POSPC=\frac{POSTA}{P}$$

where POSPC is the POS area per capita, POSTA is the POS total area, and P is the city population. Then, all maps for the study cities were generated using GIS, and these maps were examined and analyzed in regard to POS distribution and accessibility. In addition, the results of the per capita area for each city were compared against selected international standards.

To ensure the validity and reliability of the online questionnaire results and online survey questions, the questionnaire was evaluated by a group of experts, academics, and specialists from the field of urban landscape planning, with one from Bahrain, two from the UK, and three from Saudi Arabia. This group was asked for their opinions on the suitability of the questionnaire, and had the opportunity to add to, delete, or reformulate the questions. The recommendations by these experts included the following suggestions: The first version of the questionnaire did not ask where users came from (i.e., which district in each city), but this information would show the distance that users were taking to reach the study sites. In addition, three of the experts suggested asking the users in the online survey if they used other spaces at a city-scale. All comments and suggestions by the experts were taken into account, and both the online survey and on-site interview questions were amended accordingly. 

Internal consistency means that each paragraph of the questionnaire is consistent with the axis that is relevant to that specific paragraph. Correlation coefficients were calculated for each paragraph, and the total score of the axis was used to verify the validity of the questionnaire. In general, reliability means the degree to which an instrument gives the same result each time it is used in the same conditions with the same subjects. There are many methods of ascertaining reliability. In this study, Cronbach’s Alpha was used to calculate the reliability of the data collected through the online questionnaire.

## 3. Results

The results section is divided into two parts. The first part presents and discusses the online survey and the semi-structured interview results and provides a comparison with the literature. The second part presents the POS area per capita in each of the cities studied. This part also identifies the level of accessibility of the POS compared to international and MoMRA standards. It also discusses the monetary value of each open space to support the proposed planning approach of the study. Ultimately, based on the results, we propose a POS planning approach for Saudi Arabia.

### 3.1. User Preferences for Public Open Spaces and Patterns of Use

#### 3.1.1. Online Survey Results

The total number of participants in the online survey was 1596. Of these, 744 were from Jeddah, 670 were from Riyadh, and 182 were from Dammam (80 males and 102 females, comprising 174 Saudis and eight non-Saudis). The total number of male participants was 1112, or 70%, and 30% of the participants were female. The majority of the participants were Saudis, at 95%, as shown in Appendix A (Table A4).

Across the three cities, 54% of the participants said that POS were available near their place of residence (300 m or a 5 minutes’ walk away), with 55%, 53%, and 74% in Jeddah, Riyadh, and Dammam, respectively. Further, 46% of the participants clarified that they did not have access to nearby POS. When asked whether they used these gardens and parks, 40% in the three cities did not use these spaces for due to issues associated with design, quality, maintenance, safety, accessibility, a lack of planting and water features, or a lack of activities. Other stated reasons included site hygiene and user behavior. On the other hand, 37% sometimes used and 24% often used the nearby POS for physical activity, mental wellbeing, gathering, and to escape from everyday routines. In addition, 10% of those with POS nearby used them for an unknown reason. In Jeddah, only 16% used nearby spaces, compared with 29% in Riyadh and 34% in Dammam, as shown in Appendix A (Table A5).

When the participants were asked if they had access to POS at the neighborhood level, 1147 responded “yes”, 428 answered “no”, and 21 did not know: 24% used these gardens or parks, 36% did not use them, and 40% sometimes used them. The responses of the participants in each city regarding their use of POS in their neighborhood. In Jeddah, Riyadh, and Dammam, 51%, 26%, and 31%, respectively, did not use their neighborhood POS. The percentage of those using the neighborhood POS was low for the same reasons mentioned previously. The majority of those using these POS did so for physical activity in all cities. For POS on a city scale, 66% reported using these spaces, 22% sometimes used them, and 11% did not use them. In Jeddah, Riyadh, and Dammam, 79%, 51%, and 69%, respectively, used the various POS, such as waterfronts and parks. A minority in these cites did not report using the city-level POS, with 6%, 19%, and 7%, respectively. The majority of the participants in the three cities used these POS for picnicking (59%), mental wellbeing (53%), and physical activity (47%). In addition, 37% used the city-level POS for family gatherings, and 33% used them to escape from everyday routines, as can be seen in Appendix A (Table A6).

To understand user preferences for POS use, the last section of the online questionnaire used a Likert scale to measure how users perceived the POS [92]. The most highly ranked opinions in all cities were “maintenance plays a role in my use of POS in general (4.76),” and “the design attracts me to the POS (4.67),” followed by “I enjoy the journey to the POS (4.60),” “I do not feel like using the gardens in my area (4.59),” “I prefer to use gardens and parks in large open areas (4.14),” and “I prefer to use gardens and parks away from my home (4.06).” The results of the online survey support the study argument for shifting the focus on the provision of parks and gardens away from the neighborhood scale only toward creating a city-scale POS system. This is because users consider the journey to the POS as part of the experience. The following statements are related to other aspects of POS and user attitudes: “a lack of a sense of security affects my use of the POS (3.86),” “the more users that are present in the site, the more I use that site (3.71),” “vegetation and planting attract me to the POS (3.58),” “site elements attract me to the POS (3.39),” “water bodies attract me to the POS (3.32),” “customs and tradition affect how I use the POS (2.20),” and “I do not use POS because I don’t feel accepted socially (2.09).” Further details are shown in Appendix A (Table A7). These findings support previous studies that relate to perceptions of POS. 

#### 3.1.2. Semi-Structured Interview Results

A total of 917 interviews were conducted (Jeddah, 336; Riyadh, 284; Dammam 297): 55% of the interviewees were Saudi and 45% were non-Saudi, with 54% male and 46% female interviewees (Appendix A (Table A8)) which also shows the age distribution of the interviewees at the study sites. 

At the Jeddah waterfront, the majority of the interviewees visited daily (73%). Most expressed feelings regarding their daily visits being due to “the design,” “the plantings,” “the open area,” and “the facilities.” It is important to note that the selected area on the waterfront was opened to the public in 2018. This part of the waterfront has since become the main destination for Jeddah residents and visitors. One of the male Saudi interviewees stated that “this corniche has become a vibrant part of Jeddah in the day and night, packed with people of different ages, genders, and nationalities.” A non-Saudi female said, “I live more than 40 minutes’ drive from the corniche, and I just do it every day, I do not know why.” A Saudi female said, “this place is magnificent. I wish we had more and more of these spaces in Jeddah.” The interviewees were asked how they reached the site. The responses included driving a private car, walking, or public transportation (taxi or others), at 80%, 19%, and 2%, respectively.

When the interviewees were asked about the availability of gardens and parks near their homes or within their neighborhoods, 57% confirmed that they had a nearby POS, 40% stated that there was no POS nearby, and 3% did not know if there was a POS close to their homes. While 190 out of 336 interviewees did have POS close to their homes, only 4% used these spaces. One of the interviewees commented that “I do not like to use it because it’s empty.” In addition, another Saudi interviewee stated that “I have been living in my neighborhood for more than 20 years, and I rarely see someone use the garden. It’s well maintained, and I see the municipal team trimming trees, and mowing grass, and fixing the lighting, but still no one uses it.” A migrant from Jordan stated that “my kids hate it when I take them to the garden near our home. They love to ride in the car and come here even for one-hour play.” His wife also commented that “even if driving to the waterfront takes more than 30 min, they never get bored.” It is important to note that the majority of the interviewees (Saudis and migrants) with access to a nearby POS described the space as follows: “empty,” “boring to use the nearby park,” “I see it every day, and I do not feel like using it,” “my kids do not like it,” “regardless of the busy traffic in Jeddah, we do it every day while we have one in front of our home,” and, “I want to be away from home.” 

The analysis of interviewee responses regarding the reason they used the waterfront, revealed that the majority used it for family gatherings, picnics, and physical activity. A Saudi female stated that “my grandmother likes this place, and every time my mother asks her to go out, she picks this waterfront, and we have a small garden near our house.” The majority of the migrant interviewees stated that “this place has become the destination for a picnic all the time, since it opened, we just love to use it, and every time we have a relative visiting, we bring them here.” Jeddah has various walkways distributed in its different districts and close to neighborhoods. However, the main sports track on the waterfront has become one of the main destinations in Jeddah. Other responses regarding why the interviewees used the waterfront included mental wellbeing, meeting friends, and escaping from everyday stress. These responses were repeated 117, 69, and 66 times, respectively. In addition, nine interviewees had no particular reason for visiting.

In Al Salam Park in Riyadh, 72 (25%) of the interviewees visited the park daily for physical activity, and 112 (39%) used it weekly for picnicking and family gatherings. In addition, 23% used it monthly, and 12% used it once or twice a year. Like the interviewees in Jeddah, the majority reached the park using a car (89%), while some (7%) walked because they lived nearby (15 to 20 min). Around 74.6% of the interviewees had a POS near their homes. However, only 4% used these spaces. 

The interviewee statements regarding these spaces were somewhat similar to those from Jeddah. A Saudi male who came every day to exercise stated that “there is a linear park near my home, and I have no reason why I drive 20 min in Riyadh traffic to walk here. I just do not feel like walking near my house.” He also added, “sometimes when I am busy or have a commitment, I walk close to my home, but I do not feel like I did exercise, I keep thinking of this park and imagine myself walking there.” Similarly, 25% of the interviewees agreed that they did not walk or exercise near their homes because the POS were “empty,” and when they walked in Al Salam Park, they felt that other people who walked “encouraged” and “motivated” them because “walking alone is boring.” 

The majority of the interviewees visited this park for physical activity (84%). In addition, the majority of migrants used the park for picnics and family gatherings. An Indian male stated that “we come weekly, we enjoy it, the green, the lake, everything is nice.” This interviewee lived far from the park (a 30-min drive) and brought his family by taxi. When he was asked about the cost, he replied, “yes, it is costly, but I must entertain my kids and family, and we spend a long time, so it is worth it.” The park design concept was built to represent the architectural heritage and nature of Saudi Arabia, which is why the Saudi visitors and some migrants regard it as “magnificent” to see the “natural stones used in building the park” and the “mature date palms.” In addition, Riyadh is an arid city and the water in this park makes it “alive” for both Saudis and migrants. 

The waterfront in Dammam was used daily by 71% of the interviewees, 15% weekly, 9% several times per year, and 5% once or twice per year. Around 86% visited the site using a private car, which is similar to the results for Jeddah and Dammam. As described for the sites in Jeddah and Riyadh, the majority of the interviewees (236) had nearby gardens and parks, but only 5% used them. The reasons expressed by the interviewees matched the responses for the Jeddah waterfront and Al Salam Park in Riyadh. However, a Saudi female further said, “I do not feel comfortable walking in the neighborhood, even in the daytime,” Another migrant female said, “walking alone is not safe.” A Saudi male stated that “the government spent good money to build parks and gardens, but we do not use them, so it is a waste of money.” A further discussion took place with this individual, as he is a planner and shares a similar perspective to the authors on the POS provision in Saudi Arabia.

A total of 245 interviewees at the Dammam waterfront used the space for physical activity. A Saudi male said, “I like exercising near the sea, it is good for my health.” In addition, the interviewees felt that walking and exercising close to the sea was “good for my health,” “good for my mind,” and “helps me to escape.” In addition, the selected part of the waterfront in Dammam is a very “popular place in Dammam, and many people come from nearby cities such as Bahrain to visit their family and friends, and they always come to the sea.” An Egyptian female added, “when I feel angry, I always drive by the corniche. I like it, I feel relieved and relaxed.” 

### 3.2. Public Open Spaces Per Capita and Accessibility Measures

International standards suggest certain values per capita for POS: The World Health Organization, 9 m^2^; Public Health Bureau USA, 18 m^2^; European Union, 26 m^2^; and United Nations standards, 30 m^2^. Comparing the results of this study with these standards indicates whether or not there is a shortfall in each city. In addition, the accessibility of POS is discussed based on local and international standards. To support the study argument, this section presents how much the Saudi government has invested in POS provision and the economic costs of these spaces. 

#### 3.2.1. Riyadh Public Open Spaces Per Capita

**Figure 3:**
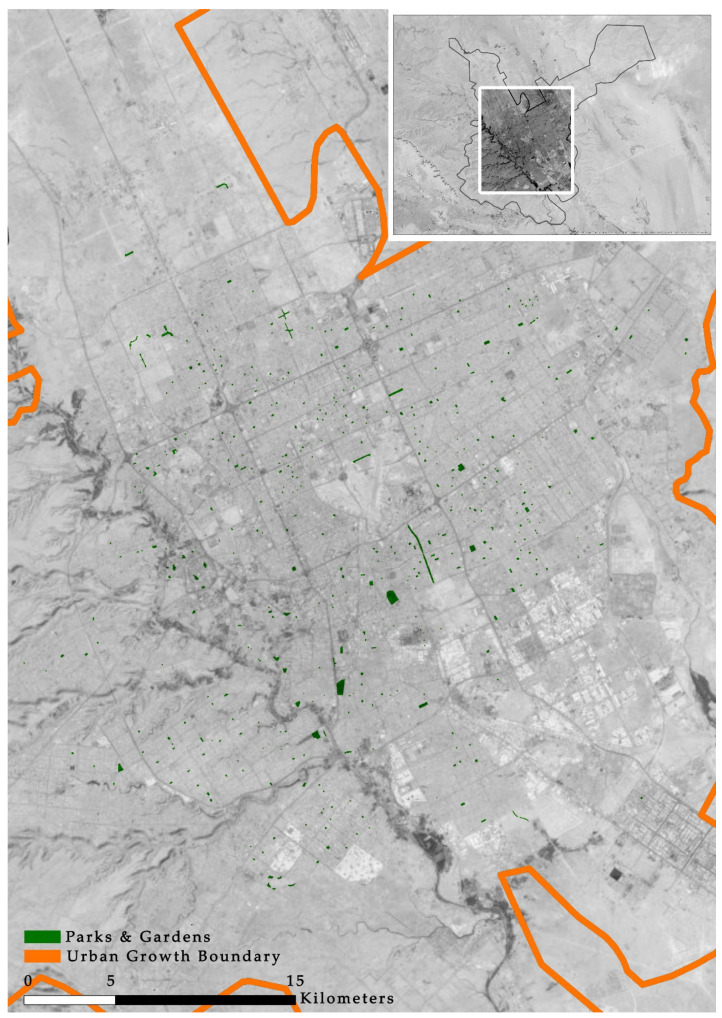
Public open space distribution in Riyadh.

As mentioned previously, all gardens and parks located at the neighborhood, district, or city levels were drawn using a GIS to identify their locations and areas. Riyadh has 450 gardens and parks that are distributed as shown in Figure 3. The total area of these gardens and parks is 6,161,567 m^2^. This means that there is one POS for every 11,637 inhabitants. 

By applying the equation mentioned in the methodology section, we find that there is 1.18 m^2^ of POS per capita in Riyadh. This figure is below all international standards. Table 1 compares POS per capita in Riyadh with the World Health Organization, Public Health Bureau USA, European Union, and United Nations standards. To meet WHO standards, for example, the city would need to add around 41 million m^2^ of POS, and this figure rises to 150 million m^2^ to meet United Nations standards.

#### 3.2.2. Jeddah Public Open Spaces Per Capita 

**Figure 4:**
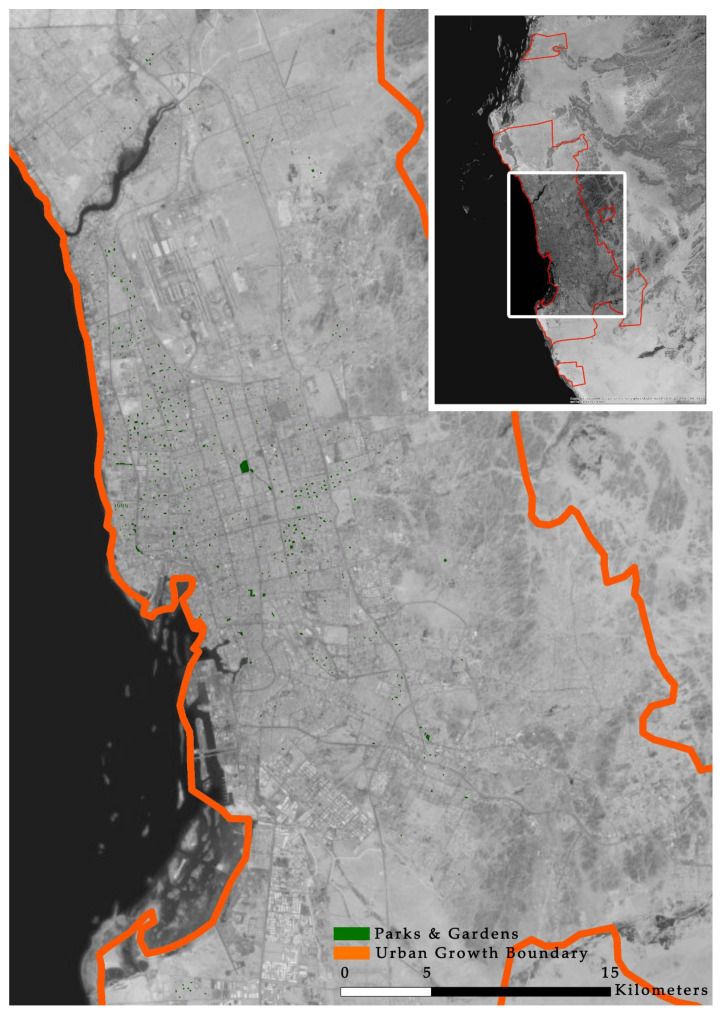
Public open space distribution in Jeddah.

Figure 4 shows the distribution of POS, generated using a GIS. Jeddah has 2,050,493 m^2^ of POS in 432 locations, which means that there is one garden for every 8949 inhabitants.

By applying the equation mentioned in the methodology section, we find that there is 0.5 m^2^ of POS per capita in Jeddah. This is a very low level when compared to international standards. Table 2 compares the POS per capita in Jeddah with the international standards used in this study. To meet European Union standards, for example, there is a need for an extra 100 million m^2^ of POS and 31 million m^2^ to meet WHO standards.

#### 3.2.3. Dammam Public Open Spaces Per Capita

**Figure 5:**
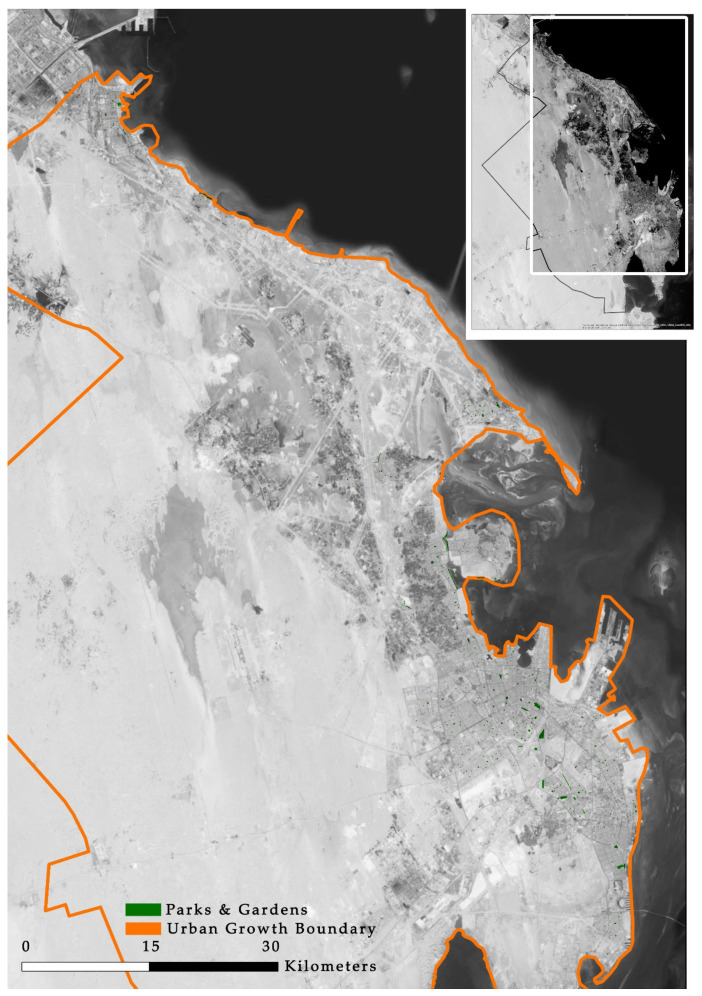
Public open space distribution in Dammam.

Figure 5 shows the distribution of POS, as generated using a GIS. Dammam has 367 gardens and parks, as shown in the figure below. The total area of these gardens and parks is 5,526,933 m^2^, which means that there is one POS for every 2791.30 inhabitants.

By applying the equation, we find that the POS per capita in Dammam is 5.4 m^2^, which is close to the WHO standard. Table 3 compares POS per capita in Dammam with the international standards used in this study. To meet WHO standards, there is a need for an extra 40% area of POS in Dammam. It is essential to highlight that Dammam’s POS per capita value is high when compared with that of Riyadh and Jeddah, and this is due to the low population numbers in Dammam.

#### 3.2.4. Public Open Space Accessibility

**Figure 6:**
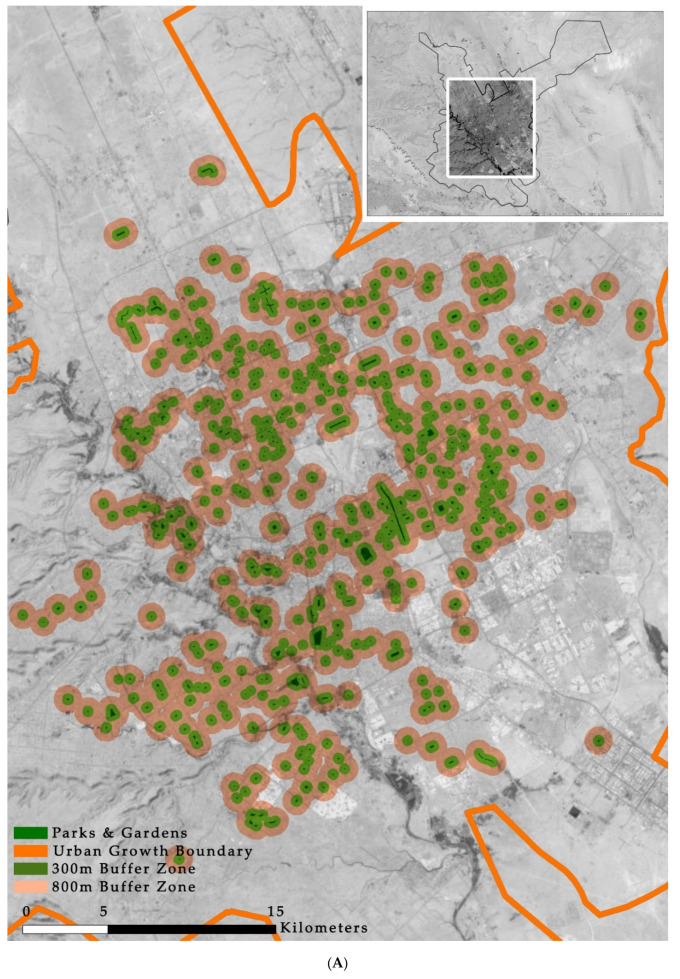

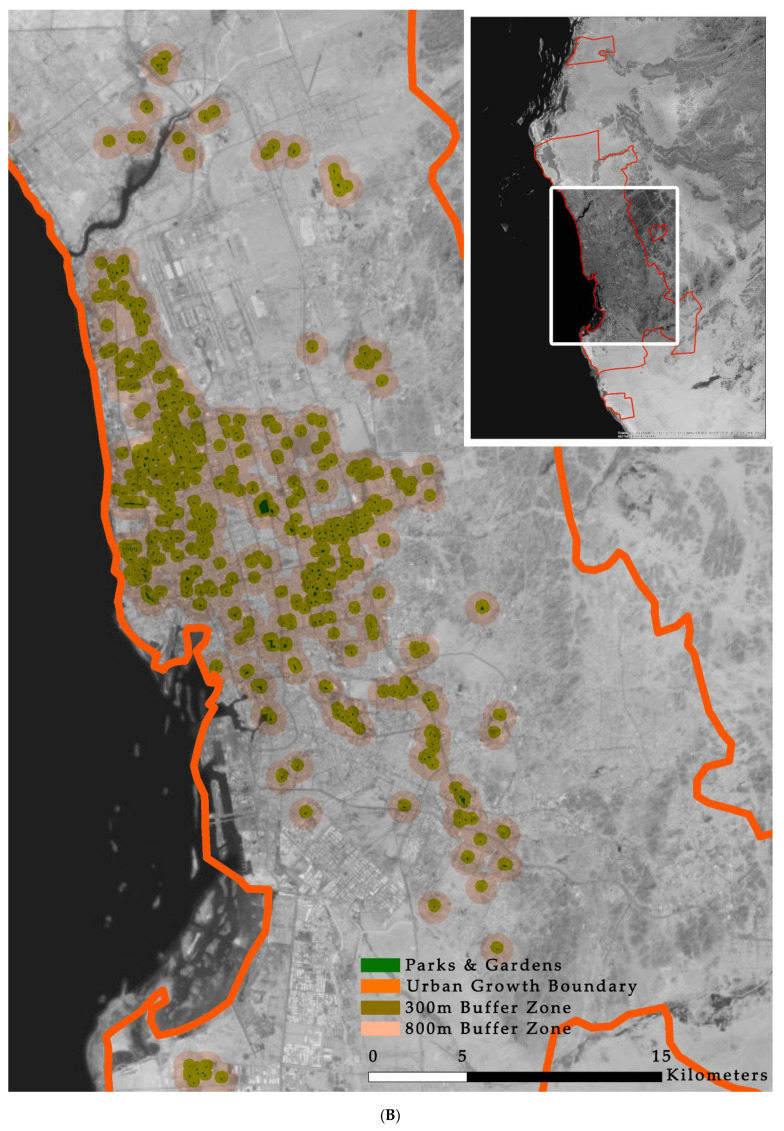

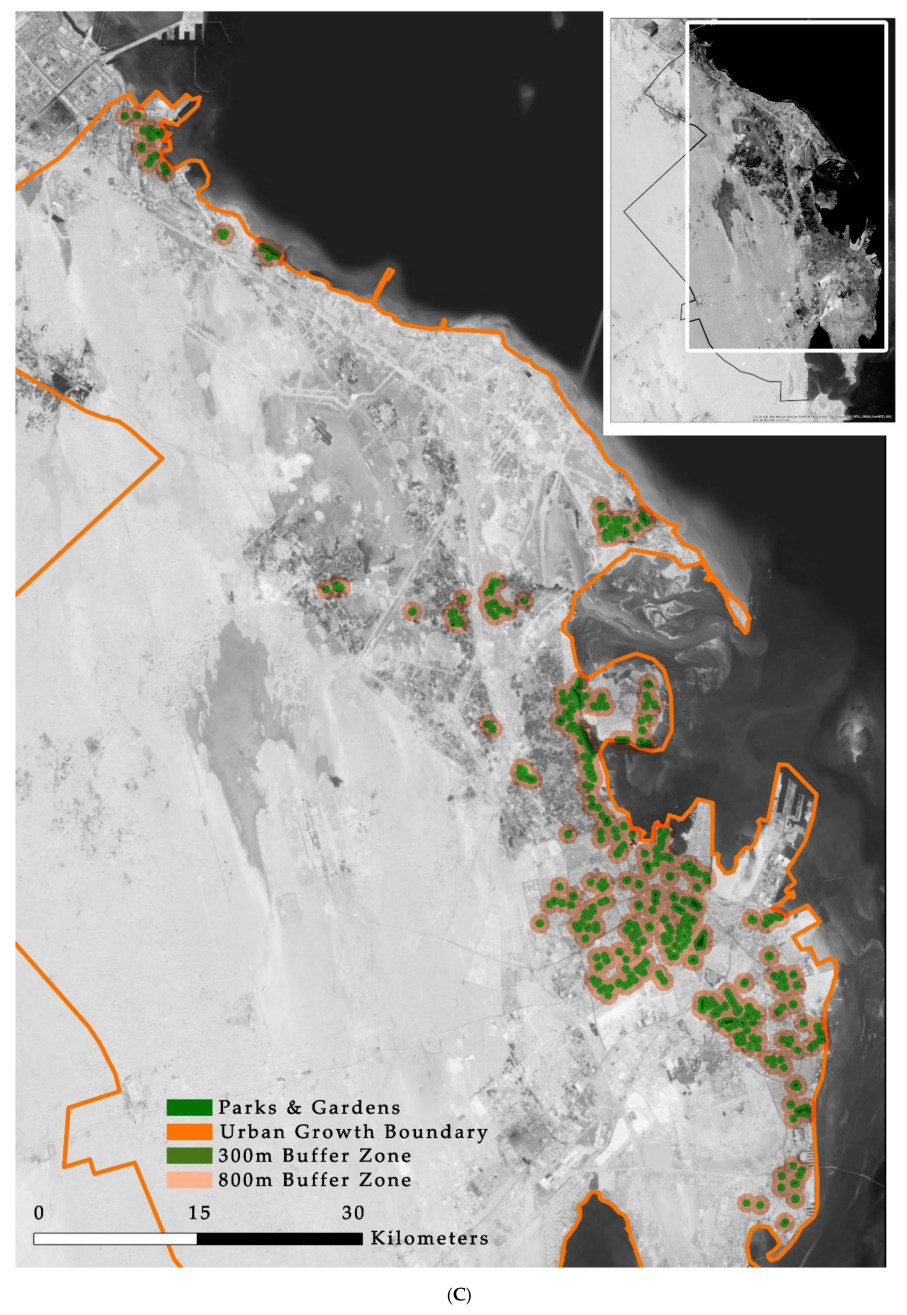
Public open space accessibility in (**A**: Riyadh, **B**: Jeddah, and **C**: Dammam).

As mentioned in the Introduction regarding the importance of POS accessibility and the various standard approaches for ensuring that POS are accessible to all residents, we measured and analyzed accessibility in the three cities to indicate the spatial planning of POS in Saudi Arabia. In Figure 6, we can see that the current POS provision approach in the three cities does not allow a sufficient level of accessibility in the city level in 300 m radius, especially in Riyadh and Dammam. 

#### 3.2.5. Public Open Spaces Monetary Value 

As mentioned in many studies, POS offer great value to residents and cities. However, very few studies have explored the costs of POS provision. The cost of these spaces is not a one-time fee, but an ongoing expense for governments around the world due to maintenance requirements. The cost of POS provision includes design and consultation fees, implementation, management, and maintenance. Maintenance in general can equate to 85%–95% of the total cost of a POS [93]. The authors obtained information from MoMRA regarding the implementation costs of neighborhood parks and gardens. The average is 250 USD or 1000 Saudi Riyal per m^2^. This information is essential to support the argument of the study and the proposed approach to POS provision in Saudi cities.

## 4. Discussion

The results of the online survey revealed that 56% (898 out of 1596) of the participants had gardens or parks within a five-minute walk or 300 m of their homes. However, 40% did not use these spaces for various reasons related to site design, maintenance, and hygiene. Only 13% used these spaces for physical activity. At the neighborhood scale, 1147 confirmed that they had access to POS. However, only 24% used the POS, 35% sometimes used them, and 36% did not use them at all. At the city level, 66% of the participants used the various POS outside their own neighborhoods. In Jeddah, Riyadh, and Dammam, 11% did not use city spaces. These findings support the argument of this study, that the minority are using POS nearby or within the neighborhood, and the majority prefer to use POS on the city-scale.

In Saudi Arabia, people enjoy traveling to POS, and consider the journey to the site to be integral to their visit. People enjoy the travel for its own sake rather than specifying a means of transport, although each method has advantages and disadvantages, such as cost and time [94]. In addition, the majority of the participants agreed with the statement “I do not feel like using the gardens in my area” because they considered the journey to be part of the experience. From the result of this study, we also found that people are not using the nearby spaces due to the design and maintenance issues of these sites.

A review of the interviewee responses reveals their perceptions of the study sites. Regardless of the route they take to reach a site, the participants enjoy the sites. Other studies have proposed that having local parks near to people’s homes encourages the use of those spaces [37], while driving to reach POS often deters their use [39]. However, the findings of this study indicate that people enjoy driving to reach POS as the journey is part of the experience. This result contradicts previous findings and supports similar studies conducted in the Saudi Arabia context for POS use [20].

It is important to highlight that the results of the online survey and the semi-structured interviews reveal a range of benefits of POS, such as mental wellbeing, physical activity, social cohesion, and evidence of place attachment. However, these findings will not be discussed in detail because they are outside the main scope of this study. Still, it is important to state that this study adds to the evidence in the international body of literature regarding POS benefits. In addition, the respondents mentioned that they did not use nearby POS for reasons such as safety and design, which is in line with a wide range of studies [95,96].

Furthermore, the presence of migrants in public open spaces, such as waterfronts and parks, has been found to be high in other studies [20,71]. However, the current study does not show that clearly. One reason for this is that when migrant users were approached by the authors while conducting semi-structured interviews, they refused to participate because of a range of issues, such as residence permit status. The authors tried their best to include non-Saudis in the sample, as some studies suggest that public open spaces have a positive impact on migrants and their sense of belonging and reviving memories.

The review of the POS per capita for Riyadh, Jeddah, and Dammam reveals that the three large cities lag far behind the four international standards that are used in this study for comparison, at 1.18, 0.5, and 5.4 m^2^ per capita, respectively. While the NTP aimed to increase the POS per capita in Saudi cities to 3.9 m^2^ by 2020, the results show that the two largest cities (Riyadh and Jeddah) are far from this target. This finding indicates an apparent lack of POS provision and understanding of the importance of POS to residents and cities. 

To achieve value from POS in an urban context, especially regarding physical activity, people should be able to access POS of at least 20,000 m^2^ [24]. We ran an area analysis of the POS in the three cities using GIS to identify the areas of gardens and parks. Table 4 shows the number of gardens in Riyadh, Jeddah, and Dammam greater or less than 20,000 m^2^. The results revealed that, in the three cities, the availability of POS greater than 20,000 m^2^ is very limited, at 13%, 2%, and 13%, respectively. The percentage was derived according to the total area of the POS in each city. In addition, the online survey analysis indicates that 56% of the respondents had POS near their residences. However, only 24% used these spaces. 

Furthermore, 41% of the respondents to the questionnaire used other spaces in the city for physical activity. The semi-structured interviews of respondents in Jeddah, Riyadh, and Dammam showed that 53%, 84%, and 83%, respectively, came to the study sites for physical activity. This finding supports the broad consensus of a previous study [24], and supports the argument of this study that MoMRA should not only apply international standards for POS provision but also needs to understand user preferences and needs.

The analysis of POS accessibility shows that the current spatial distribution of POS in the studied cities fails to provide accessible POS for all residents. Moreover, there is a lack of a national strategy for POS planning in Saudi Arabia [71,97,98]. The analysis of POS accessibility within a 300 m radius indicates that not all areas in each city are covered by POS. In addition, the MoMRA standard for accessibility fails to provide sufficient coverage of POS in the cities. Regardless of whether these POS are used, policymakers need to adopt a POS typology and create a POS network system to enhance the level of accessibility [18,29,80,98,99].

The current POS hierarchy presented in Table A2 was developed by MoMRA based on function and catchment, which is not considered as POS typology. A POS typology could provide a more grounded understanding of why POS are important for both the city and the users. It is also necessary for a meaningful understanding of POS planning, design, and management. There are various approaches to classifying and designing a POS typology, and it facilitates a better understanding of the city landscape and natural elements along with the urban context.

The results indicate consistency among the internal data, with correlation coefficient values for all statements between 0.111 and 0.581. These values were found to be significant at a 1% level, as shown in (Appendix A (Table A9)). In addition, the Cronbach’s Alpha was 0.507, as shown in (Appendix A (Table A10)).

MoMRA has made substantial investments in POS provision in Riyadh ($1,540,391,750), Jeddah ($512,623,250) and Dammam ($1,381,733,250). Importantly, these costs do not include design, consultation, or maintenance fees. Further, MoMRA is the only body financing these projects, and there is no involvement of the private sector. To achieve the WHO standard of POS per capita, which is 9 m^2^, the government would need to invest another $20 billion in POS provision. To meet the UN standard, which is the highest of the four standards, there would be the need to invest $74 billion, as shown in (Appendix A (Table A11)). These results support the proposed planning approach suggested by this study to involve the private sector in financing and implementing POS in Saudi cities.

### Criticisms and Suggestions for Saudi Arabia POS Planning

Analysis of the results shows the current practice of POS planning in Saudi cities, and highlights several problems. These problems relate to the planning approach for POS spatial distribution and the lack of understanding of residents’ needs and preferences. In addition, the responsible governmental agencies of POS planning and design show a lack of understanding of the importance of POS to the cities and residents. These issues inform the debate of this study and the proposed concept for POS planning in Saudi Arabia. 

Regarding the POS area per capita in Saudi Arabia, we found from the results that the three largest cities are far from achieving WHO standards, especially Riyadh and Jeddah. In addition, the level of POS accessibility when applying the international and MoMRA standards means that a massive area of each city’s urban context is not covered. MoMRA is trying to apply planning standards without considering the culture, lifestyle, and needs of residents in Saudi Arabia. This is because the planning process of public facilities does not include public participation and stakeholder engagement during the planning stage [100]. However, key criteria of Vision 2030 include the extent of public engagement and participation in decision-making. The MoMRA guidelines for planning, urban design, and POS provision are not tailored to fit the urban context of Saudi cities. At the same time, the Saudi government has spent billions of USD to provide POS in Saudi cities. However, neighborhood gardens are neglected by residents because they prefer to use other spaces in the city (even for everyday use), regardless of the quality of the local spaces. In addition, the findings revealed that these spaces are neglected for several reasons, such as issues related to design, maintenance, and safety. 

This study suggests that MoMRA and the relevant municipalities should pay more attention to POS spatial distributions at a city-scale and reform the guidelines on neighborhood POS planning. This study found that city-scale parks and waterfronts are more desirable to residents in Saudi Arabia than neighborhood gardens. In this regard, the spatial planning of Saudi cities needs to address such spaces, especially central parks, of which there are few in the country. Riyadh city took the initiative and introduced four large city-scale park projects: King Salman Park Project, Green Riyadh Project, Sports Boulevard Project, and the Riyadh Art Project.

This proposed concept of POS provision suggests a need for different types of public spaces in all Saudi cities. In addition, clear focus needs to be given to neighborhoods’ planning and design to develop their urban planning, walkability and streetscapes, and to introduce new types of spaces surrounding neighborhood facilities and services, such as different types of public open spaces, which are not a common type of space in Saudi Arabia. Mosques, schools, and other neighborhood facilities also have leftover spaces. These spaces, when developed and designed, can form the main POS at a neighborhood-scale, and can offer a range of gardens and benefit parks such as enhancing physical and mental wellbeing, and helping to mitigate the elevated temperature where people live, and so forth.

In addition, MoMRA needs to establish a POS system and network for Saudi cities based on each city’s urban and rural fabric. This will allow decision-makers to properly understand POS spatial planning and create a national system of POS. This step will facilitate the creation of a comprehensive POS typology for Saudi cities. This POS typology will offer different types of spaces within the neighborhoods and on the city-scale.

Furthermore, these proposed new types of space in the neighborhoods need to consider a range of issues that emerged from the findings of this study, such as maintenance, design, and safety. These issues, when considered in the planning and design stage of neighborhood spaces, will encourage use and make the neighborhood spaces livable. 

This proposal is based on findings that reflect user preferences. Current POS provision indicates that it is difficult to rely on international standards without understanding the local residents’ preferences and behaviors, such as which POS they prefer to use, when and how they use them, and what the pattern of using POS is. Furthermore, this proposal for POS planning will not adversely affect the quality of life in the relevant neighborhoods if the planning stage addresses the establishment of new spaces and enhances various aspects such as enhancing walkability and streetscape. Conversely, such a proposal will provide and enrich Saudi cities with various types of POS once they are considered in the municipal master plans and the national strategy for Saudi cities. 

Furthermore, this proposal does not suggest that there should be one place for all residents in a city [101]. Instead, it supports diverse and different types of POS at city and neighborhood scales. POS do not need to be attractive to all people in a city, as people differ based on their backgrounds and preferences, and a variety of POS would cater equally to different tastes and needs. In addition, proposing different POS themes is important at the city-scale by including vibrant and commercial spaces, local market spaces, playgrounds, and historical areas.

## 5. Conclusions

The current planning system for POS in Saudi Arabia fails, to some extent, to meet international standards due to current planning practices. This study argues that MoMRA is following international planning standards for POS provision, even though the country’s cultural characteristics are different from those of Western countries, and the lifestyles of residents cannot be compared to these countries. The government should examine the standard approaches to POS provision and planning to meet the needs of residents in Saudi Arabia. The main issue is determining how to provide sufficient POS and gain benefits from those spaces based on an understanding of users’ lifestyles and preferences. There is a need for flexible POS planning approaches tailored to each city’s characteristics and landscape types, which leads to the need for capacity-building in MoMRA and in the governmental agencies responsible for city planning and design

This study recognizes that the Saudi government has invested billions of USD in POS provision, but there is a need to invest much more to meet the international standards for POS accessibility and per capita areas. However, using semi-structured interviews and an online survey, the present study found that people tend not to use the parks and gardens close to their homes, regardless of the quality of these spaces. Enjoyment of the journey and the city urban context compel people to travel to particular POS in their city, such as large parks or waterfronts, which is the reverse of the tendency found in international studies. In addition, MoMRA needs to involve the private sector in the implementation and planning processes for POS. This involvement will have a positive impact on government spending and allow for partnerships that will benefit residents and the city. In addition, this type of partnership will help the government to control the spending on POS provision in the Saudi cities. 

The research in the literature regarding the accessibility and per capita areas of POS varies between qualitative and quantitative approaches. The proposed approach based on the findings of this study shifts the focus of POS provision from neighborhood scale to the city level, to cater for residents’ needs by creating typology and POS system and network in each city. This approach would increase the POS per capita in Saudi cities while finding a balance between city and neighborhood scale POS that improves overall financial, environmental, and social sustainability.

The proposed planning approach for POS would not adversely affect the quality of life in residential neighborhoods. Rather, the proposed planning system would focus on enhancing the urban planning of residential neighborhoods by reducing the requirements for POS provision and shifting investment to improving the streetscape, developing walkability in neighborhoods, and linking neighborhood services together. This process would introduce new types of POS, such as different types of public open spaces. The spaces around mosques, schools, and other neighborhood services will serve as the main open spaces for such neighborhoods. This proposal will have a positive impact on the Saudi cites and it will have direct and indirect impacts on the residents’ health as it will promote physical, mental, and social wellbeing.

## Figures and Tables

**Table 1:** Comparison of Riyadh POS per capita and international standards.

Organization	Standard m^2^/Capita	POS Per Capita m^2^	Required POS Area to Achieve Standard m^2^	Shortage in m^2^
World Health Organization	9	1.18	47,132,109.00	40,970,542.00
Public Health Bureau USA	18		94,264,218.00	88,102,651.00
European Union	26		136,159,426.00	129,997,859.00
United Nations	30		157,107,030.00	150,945,463.00

**Table 2:** Comparison of Jeddah POS per capita and international standards.

Organization	Standard m^2^/Capita	POS Per Capita m^2^	Required POS Area to Achieve Standard m^2^	Shortage in m^2^
World Health Organization	9	5.4	36,545,319.00	34,494,826.00
Public Health Bureau USA	18		73,090,638.00	71,040,145.00
European Union	26		105,575,366.00	103,524,873.00
United Nations	30		121,817,730.00	119,767,237.00

**Table 3:** Comparison of Dammam POS per capita and international standards.

Organization	Standard m^2^/Capita	POS Per Capita m^2^	Required POS Area to Achieve Standard m^2^	Shortage in m^2^
World Health Organization	9	5.4	9,219,681.00	3,692,748.00
Public Health Bureau USA	18		18,439,362.00	12,912,429.00
European Union	26		26,634,634.00	21,107,701.00
United Nations	30		30,732,270.00	25,205,337.00

**Table 4:** POS greater than 20,000 m^2^ in the study cities.

City	POS Number	POS > 20,000 m^2^	Percentage
Riyadh	450	61	14%
Jeddah	432	7	2%
Dammam	367	49	13%

## Appendix A

**Table A1:** List of Ministry of Municipal and Rural Affairs Guidelines.

MoMRA Guidelines
MoMRA urban design guidelines
A manual on addressing and planning public open spaces in cities
Standards for designing, constructing, and maintaining public parks
Special regulations for esthetic elements in public spaces and beaches (sculptures)
Technical specifications for ‘greening’ projects
Greening project implementation manuals
A manual on irrigation for greening projects inside cities
A manual on the selection of plants for greening projects in different environments
Guidelines for selecting plants suitable for greening projects in different climate zones (2001)
Planning standards for recreational spaces in cities
Guidelines for preparing and updating the city master plan

**Table A2:** Ministry of Municipal and Rural Affairs public open space function base hierarchy.

Public Open Spaces Type	Area (m^2^) by Density	Access Method		Catchment Distance Radius - (m)
		Walking	Car	
Pocket Parks	800–3000	x		100–200
Children Parks	900–6000	x		150–275
Residential Block Parks	4000–5000	x		200–350
Residential Block Playgrounds	3000–6000	x		250–500
Neighborhood Parks	5000–10,000	x	x	400–800
Neighborhood Sports Playgrounds	15,000–35,000	x	x	1000
District Parks	20,000–60,000		x	2500–5000
City Parks	70,000	x	x	-
Amusement Parks	2000		x	-
Camping Sites	varied		x	-
Special Parks (Waterfront, Zoo, Planetarium, Natural Landscapes, etc.)	varied		x	-
Recreation Parks	varied		x	-

Source: MoMRA guidelines for the planning standards of recreational spaces (2005), translated by the author; x = the access method applied.

**Table A3:** Geo-spatial analysis of the study cities.

City	Area km2	Weather	Average Temperature	Average Rainfall	Physical Env.	Population		
						Saudi	Non-Saudi	Growth Rate
Riyadh	1913	Dry and hot in the summer (April to September) cold in the winter (September to March)	Winter lower than 15°C Summer higher than 45°C	10–20 mm	Desert	3,153,478	2,118,513	4.1%
Jeddah	1600	Hot and dry desert climate with high humidity	28°C, but in summer temperatures can go as high as 40°C	45 mm	Desert/ Coastal	1,729,192	1,728,602	3.8%
Dammam	800	Hot and dry desert climate	Average low and high temperature of around 20°C and 34°C, respectively	54 mm	Desert/ Coastal	549,925	364,568	2.3%

**Table A4:** Participant distributions according to gender, age and nationality.

	Jeddah	Riyadh	Dammam	Total	Percent
Male	560 (75%)	472 (70%)	80 (44%)	1112	70%
Female	184 (25%)	198 (30%)	102 (56%)	484	30%
Saudi	696 (94%)	646 (96%)	174 (95%)	1516	-
Non-Saudi	48 (6%)	24 (4%)	8 (5%)	80	-
18–24 years	79	97	20	196	12%
25–34 years	186	139	44	369	23%
35–44 years	68	88	39	195	12%
45–54 years	234	194	32	460	29%
Above 54 years	177	152	47	376	24%

**Table A5:** The participants’ responses regarding the use of nearby public open spaces, neighborhood public open spaces and the city scale public open spaces in the three cities.

	Respond	Jeddah	Riyadh	Dammam
Nearby POS use	Used	64 (16%)	104 (29%)	46 (34%)
	Unused	224 (55%)	100 (28%)	32 (24%)
	Occasionally used	122 (30%)	150 (32%)	56 (33%)
Neighborhood POS use	Used	96 (23%)	114 (22%)	62 (34%)
	Unused	224 (51%)	132 (26%)	56 (31%)
	Occasionally used	122 (28%)	236 (52%)	40 (36%)
City-scale POS use	Used	588 (79%)	344 (51%)	126 (69%)
	Unused	42 (6%)	126 (19%)	12 (7%)
	Occasionally used	114 (15%)	200 (30%)	44 (24%)

**Table A6:** The reasons for using city-level public open spaces.

Reason	Jeddah	Riyadh	Dammam	Total Responses
Friend gatherings	14	18	4	36 (2%)
No reason	30	42	4	76 (5%)
Escape from everyday routine	314	144	68	526 (33%)
Family gatherings	328	176	78	582 (37%)
Physical activity	388	264	98	750 (47%)
Mental wellbeing	484	242	112	838 (53%)
Picnics	490	324	120	934 (59%)

**Table A7:** Internal consistency of the online survey statements.

Item	SD	D	N	A	SA	Mean	Std. D
Maintenance plays a role in my use of POS in general	20	8	46	188	1334	4.76	0.649
The design attracts me to the POS	24	10	110	182	1270	4.67	0.760
I enjoy the journey to the POS	24	24	104	266	1178	4.60	0.801
I do not feel like using the gardens in my area	14	14	146	260	1162	4.59	0.763
I prefer to use gardens and parks in large open areas	78	36	296	360	826	4.14	1.102
I prefer to use gardens and parks away from my home	60	96	322	332	786	4.06	1.127
A lack of a sense of security affects my use	174	126	250	246	800	3.86	1.392
The more users that are at a site, the more I use it	82	166	472	292	584	3.71	1.207
Vegetation and planting attract me to the POS	180	250	326	148	692	3.58	1.450
Site elements attract me to the POS	196	154	524	270	452	3.39	1.318
Water bodies attract me to the POS	238	248	386	208	516	3.32	1.440
Customs and tradition affect how I use POS	780	224	268	142	182	2.20	1.414
I do not use POS because I do not feel accepted socially	856	200	248	124	168	2.09	1.394

SA = Strongly Agree; A = Agree; N = Neutral; D = Disagree; SD = Strongly Disagree; Std. D = Standard Deviation.

**Table A8:** The interviewees’ gender, nationality, and age details at the study sites in the three cities.

	Jeddah	Riyadh	Dammam	Total	Percent
Saudi Male	106	85	87	278	-
Saudi Female	79	87	58	224	-
Non-Saudi Male	95	64	59	218	-
Non-Saudi Female	56	48	93	197	-
Saudi	185	172	145	502	-
18–24 years old	43	43	39	125	25%
25–34 years old	27	35	36	98	19%
35–44 years old	49	29	35	113	23%
45–54 years old	43	49	24	116	23%
Above 54 years old	23	16	12	51	10%
Non-Saudi	151	112	152	415	-
18–24 years old	19	22	25	66	16%
25–34 years old	40	27	33	100	24%
35-44 years old	49	31	55	135	33%
45–54 years old	23	20	24	67	16%
Above 54 years old	20	12	14	46	11%

**Table A9:** Internal consistency of the online survey statements.

	Item	Corr. Coefficient
1.	Water bodies attract me to the POS	0.111 **
2.	I prefer to use gardens and parks in large open areas	0.413 **
3.	Site elements attract me to the POS	0.352 **
4.	Vegetation and planting attract me to the POS	0.237 **
5.	The design attracts me to the POS	0.495 **
6.	Maintenance plays a role in my use of POS in general	0.509 **
7.	I enjoy the journey to the POS	0.581 **
8.	The more users at the site, the more I use it	0.529 **
9.	I do not feel like using the gardens in my area	0.537 **
10.	I prefer to use gardens and parks away from my home	0.497 **
11.	A lack of sense of security affects my use	0.484 **
12.	I do not use POS because I do not feel accepted socially	0.360 **
13.	Customs and tradition affect how I use POS	0.305 **

** Significant at the 0.01 level. Corr. Coefficient = correlation coefficient

**Table A10:** The value of Cronbach’s Alpha.

Number of items	Cronbach’s Alpha
13	0.507

**Table A11:** Costs to meet the POS per capita for each international standard in Riyadh, Jeddah, and Dammam.

	Riyadh	Jeddah	Dammam
**Current spend (250 $/m^2^)**	$1,540,391,750.00	$512,623,250.00	$1,381,733,250.00
**World Health Organization**	$10,242,635,500.00	$8,623,706,500.00	$923,187,000.00
**Public Health Bureau USA**	$22,025,662,750.00	$17,760,036,250.00	$3,228,107,250.00
**European Union**	$32,499,464,750.00	$25,881,218,250.00	$5,276,925,250.00
**United Nations**	$37,736,365,750.00	$29,941,809,250.00	$6,301,334,250.00

## References

[1] Klaufus C., Lindert P.V., Noorloos F.V., Steel G. (2017). All-Inclusiveness versus exclusion: Urban project development in Latin America and Africa. Sustainability.

[2] Madureira H., Nunes F., Oliveira J.V., Madureira T. (2018). Preferences for Urban Green Space Characteristics—A Comparative Study in Three Cities of Portuguese. Environments.

[3] Rahman K.M.A., Zhang D. (2018). Analyzing the level of accessibility of public urban green spaces to different socially vulnerable groups of people. Sustainability.

[4] Chen J., Chang Z. (2015). Rethinking Urban Green Space Accessibility: Evaluating and Optimizing Public Transportation System through Social Network Study in Megacities. Landsc. Urban. Planing.

[5] Arnberger A., Eder R. (2015). Are urban visitors’ general preferences for green-spaces similar to their preferences when seeking stress relief?. Urban. For. Urban. Green..

[6] Coolen H., Meesters J. (2012). Private and public green spaces: Meaningful but different settings. J. Hous. Built Environ..

[7] Francis M. (1995). Childhood’s Garden: Memory and Meaning of Gardens. Children’s Environ..

[8] Dunnett N., Qasim M. (2000). Perceived Benefits to Human Well-being of Urban Gardens. HortTechnology.

[9] Bishop J.C., Curtis M., Opie I. (2001). Play Today in the Primary School Playground.

[10] Armstrong N. (1993). Promoting Physical Activity in Schools. Health Visit..

[11] Beveridge C., Rocheleau P. (2005). Frederick Law Olmsted: Designing the American Landscape.

[12] Bundred P., Kitchinerb D., Buchanc I. (2001). Prevalence of overweight and obese children between 1989 and 1998: Population based series of cross sectional studies. Br. Med. J..

[13] Chandler T. (1978). The man-modified climate of towns. Built Environ. Environ. Man.

[14] Noble D.G., Bashford R.I., Baillie S.R. (2000). The Breeding Bird Survey 1999.

[15] Danzer G.A. (1997). Public Places: Exploring Their History.

[16] Rugg J. (2010). Defining the place of burial: What makes a cemetery?. Mortality.

[17] Somper J.P. (2001). Market Research: Property Values and Trees Feasibility Study.

[18] Karade R.M., Kuchi V.S., Salma Z. (2017). The role of green space for sustainable landscape development in urban areas. Acta Hortic..

[19] Matsuoka R.H., Kaplan R. (2008). People needs in the urban landscape: Analysis of Landscape and Urban Planning contributions. Landsc. Urban. Plan..

[20] Addas A. (2015). Motivation and Attachment in the Use of Public Open Spaces in Jeddah, Saudi Arabia.

[21] Ulrich R.S., Simons R.F., Losito B.D., Fiorito E., Miles M.A., Zelson M. (1991). Stress recovery during exposure to natural and urban environments. J. Environ. Psychol..

[22] Gražulevičienė R., Andrušaitytė S., Dėdelė A., Gražulevičius T., Valius L., Kapustinskienė V., Bendokienė I. (2020). Environmental Quality Perceptions and Health: A Cross-Sectional Study of Citizens of Kaunas, Lithuania. Int. J. Environ. Res. Public Health.

[23] Maas J., Verheij R.A., Groenewegen P.P., Vries S.d., Spreeuwenberg P. (2006). Green space, urbanity, and health: How strong is the relation?. J. Epidemiol. Community Health.

[24] Coombes E., Jones A.P., Hillsdon M. (2010). The relationship of physical activity and overweight to objectively measured green space accessibility and use. Soc. Sci. Med..

[25] Barrera F.d.l., Reyes-Paecke S., Banzhaf E. (2016). Indicators for green spaces in contrasting urban settings. Ecol. Indic..

[26] Sugiyama T., Thompson C.W. (2008). Associations between characteristics of neighbourhood open space and older people’s walking. Urban. For. Urban. Green..

[27] Gong C., Chen J., Yu S. (2013). Biotic homogenization and differentiation of the flora in artificial and near-natural habitats across urban green spaces. Landsc. Urban. Plan..

[28] Addas A., Goldblatt R., Rubinyi S. (2020). Utilizing Remotely Sensed Observations to Estimate the Urban Heat Island Effect at a Local Scale: Case Study of a University Campus. Land.

[29] Maruani T., Amit-Cohen I. (2007). Open space planning models: A review of approaches and methods. Landsc. Urban. Plan..

[30] Thompson C.W. (2011). Linking landscape and health: The recurring theme. Landsc. Urban. Plan..

[31] Dave S. (2011). Neighbourhood density and social sustainability in cities of developing countries. Sustain. Dev..

[32] Talen E. (2003). Neighborhoods as Service Providers: A Methodology for Evaluating Pedestrian Access. Environ. Plan. B Urban. Anal. City Sci..

[33] LaRosa D. (2014). Accessibility to greenspaces: GIS based indicators for sustainable planning in a dense urban context. Ecol. Indic..

[34] El-Geneidy A.M., Levinson D.M. (2006). Access to Destinations: Development of Accessibility Measures.

[35] Halden D., Jones P.L., Wixey S. (2005). Accessibility analysis literature review, measuring accessibility as experienced by different socially disadvantage groups, Working Paper 3. Computer Science.

[36] Ferré M.B., Guitart A.O., Ferret M.P. (2006). Children and playgrounds in Mediterranean cities. Child. Geogr..

[37] Krenichyn K. (2006). The only place to go and be in the city: Women talk about exercise, being outdoors, and the meanings of a large urban park. Health Place.

[38] Lloyd K., Burden J., Kiewa J. (2008). Young Girls and Urban Parks: Planning for Transition Through Adolescence. J. Park Recreat. Adm..

[39] Henderson K.A., Neff L.J., Sharpe P.A., Greaney M.L., Royce S.W., Ainsworth B.E. (2001). It Takes a Village to Promote Physical Activity: The Potential for Public Park and Recreation Departments. J. Park Recreat. Adm..

[40] Griffin S.F., Wilson D.K., Wilcox S., Buck J., Ainsworth B.E. (2008). Physical Activity Influences in a Disadvantaged African American Community and the Communities Proposed Solutions. Health Promot. Pract..

[41] Rigolon A., Browning M.H.E.M., Lee K., Shin S. (2018). Access to Urban Green Space in Cities of the Global South: A Systematic Literature Review. Urban. Sci..

[42] Rigolon A. (2016). A complex landscape of inequity in access to urban parks: A literature review. Landsc. Urban. Planing.

[43] Ewing R. (1997). Is Los Angeles-Style Sprawl Desirable?. J. Am. Plan. Assoc..

[44] Lynch K. (1981). A Theory of Good City Form.

[45] Jacobs A., Appleyard D. (1987). Toward an Urban Design Manifesto. J. Am. Plan. Assoc..

[46] Crompton J.L. (2001). The Impact of Parks on Property Values: A Review of the Empirical Evidence. J. Leisure Res..

[47] Goheen P.G. (1998). Public space and the geography of the modern city. Prog. Hum. Geogr..

[48] Brown G. (2008). A Theory of Urban Park Geography. J. Leisure Res..

[49] Witten K., Hiscock R., Pearce J., Blakely T. (2008). Neighbourhood Access to Open Spaces and the Physical Activity of Residents: A National Study. Prev. Med..

[50] Kaczynski A.T., Havitz M.E. (2009). Examining the Relationship between Proximal Park Features and Residents’ Physical Activity in Neighborhood Parks. J. Park Recreat. Adm..

[51] Sugiyama T., Francis J., Middleton N.J., Owen N., Giles-Corti B. (2010). Associations between recreational walking and attractiveness, size, and proximity of neighborhood open spaces. Am. J. Public Health.

[52] Organization W.H. (2017). Urban. Green Spaces: A Brief for Action.

[53] Natural England (2010). Nature Nearby: Accessible Natural Greenspace Guidance.

[54] Pickard B.R., Daniel J., Mehaffey M., Jackson L.E., Neale A. (2015). EnviroAtlas: A new geospatial tool to foster ecosystem services science and resource management. Ecosyst. Serv..

[55] Ståhle A. (2010). More green space in a denser city: Critical relations between user experience and urban form. Urban. Des. Int..

[56] Lancaster R.A. (1983). Recreation, Park and Open Space Standards and Guidelines.

[57] Harrison C., Burgess J., Millward A., Dawe G. (1995). Accessible Natural Greenspace in Towns and Cities: A Review of Appropriate Size and Distance Criteria.

[58] Ngom R., Gosselin P., Blais C. (2016). Reduction of disparities in access to green spaces: Their geographic insertion and recreational functions matter. Appl. Geogr..

[59] Jacobs J. (1961). The Death and Life of Great American Cities.

[60] Harun N.Z., Mansor M., Said I. (2013). The Experience of Diversity in Open Spaces of Two Historical Towns in Malaysia. Procedia Soc. Behav. Sci..

[61] Tveit M., Ode Å., Fry G. (2007). Key concepts in a framework for analysing visual landscape character. Landsc. Res..

[62] Gold S. (1972). Nonuse of Neighborhood Parks. J. Am. Inst. Plan..

[63] General Authority for Statistics (2019). Saudi Arabia Population Statistics.

[64] Fakeeh M.S. (2010). Saudization as a Solution for Unemployment: The Case of Jeddah Western Region.

[65] Mandeli K.N. (2011). Public Spaces in a Contemporary Urban Environment: Multi-Dimensional Urban Design Approach for Saudi cities.

[66] Khalifa H.K. (2001). Changing Childhood. Saudi Arabia: A Historical Comparative Study of Three Female Generations.

[67] Allan G. (1991). Family Life: Domestic Roles and Social Organization.

[68] Khalil A. (1994). Muslim Cities as a Pattern of Relationships: House-Mosque Relationship.

[69] Saleh M.A.E. (1997). Privacy and communal socialization: The role of space in the security of traditional and contemporary neighborhoods in Saudi Arabia. Habitat Int..

[70] Addas A. (2018). Landscape Architecture and the Saudi Arabia Quality of Life Program. Emir. J. Eng. Res..

[71] Addas A. (2020). Enhanced Public Open Spaces Planning in Saudi Arabia to Meet National Transformation Program Goals. Current Urban Studies. Curr. Urban. Stud..

[72] Alharbi T.H. (1989). The Development of Housing in Jeddah: Changes in Built Form from the Traditional to the Modern.

[73] Sidky T., Bastawisi A. Planning and Treatment of Urban Spaces within the Layout of Urban Expansion of the City. Proceedings of the First Arab Housing Conference—Construction Sustainability in the Arab Region, Especially the Desert Environment.

[74] Bokhari A. (1978). Jeddah: A Study in Urban Formation.

[75] Akbar J. (1984). Responsibility and the Traditional Muslim Built Environment.

[76] Alfahad F. (2008). Following a Study on the Reasons Why Residents Do Not Use Gardens and Parks Al Majmaah Municipality Established a Program to Rehabilitate (20) Parks within Neighborhoods.

[77] Al-Hathloul S., Mughal M.A. (1999). Creating identity in new communities: Case studies from Saudi Arabia. Landsc. Urban. Planing.

[78] Saleh M.A.E. (2002). The transformation of residential neighborhood: The emergence of new urbanism in Saudi Arabian culture. Build. Environ..

[79] Pigram J. (1983). Outdoor Recreation and Resource Management.

[80] Addas A., Alserayhi G. (2020). Quantitative Evaluation of Public Open Space per Inhabitant in the Kingdom of Saudi Arabia: A Case Study of the City of Jeddah. Sage Open.

[81] Tobi H., Brink A.v.d., Brink A.v.d., Bruns D., Tobi H., Bell S. (2017). A process approach to research in landscape architecture In Research in Landscape Architecture: Methods and Methodology.

[82] Kallus R. (2001). From Abstract to Concrete: Subjective Reading of Urban Space. J. Urban. Des..

[83] Kahana E., Lovegreen L., Kahana B., Kahana M. (2003). Person, Environment, and Person-Environment Fit as Influences on Residential Satisfaction of Elders. Environ. Behav..

[84] Vaus D.D. (2001). Research Design in Social Research.

[85] Cullen G. (1995). The Concise Townscape.

[86] Nicholls S. (2001). Measuring the accessibility and equity of public parks: A case study using GIS. Manag. Leisure.

[87] Moseley D., Marzano M., Chetcuti J., Watts K. (2013). Green networks for people: Application of a functional approach to support the planning and management of greenspace. Landsc. Urban. Planing.

[88] General Authority for Statistics (2015). Services guide for Riyadh.

[89] AlGarawi A. (2016). Global City Focus Jeddah.

[90] General Authority for Statistics (2010). The Preliminary Results of General Authority for Statistics of Population and Housing for the Year 2010.

[91] Tracy S.J. (2013). Qualitative Research Methods: Collecting Evidence, Crafting Analysis, Communicating Impact.

[92] Joshi A., Kale S., Chandel S., Pal D.K. (2015). Likert Scale: Explored and Explained. Br. J. Appl. Sci. Technol..

[93] McPherson G., Simpson J.R., Peper P.J., Maco S.E., Xiao Q. (2005). Municipal Forest Benefits and Costs in Five US Cities. J. For..

[94] Price L., Matthews B. (2013). Travel time as quality time: Parental attitudes to long distance travel with young children. J. Transp. Geogr..

[95] Hillsdon M.M., Panter J.R., Foster C., Jones A.P. (2006). The relationship between access and quality of urban green space with population physical activity. Public Health.

[96] Turel H.S., Yigit E.M., Altug I. (2007). Evaluation of elderly people’s requirements in public open spaces: A case study in Bornova District (Izmir, Turkey). Build. Environ..

[97] Mandeli K. (2019). Public space and the challenge of urban transformation in cities of emerging economies: Jeddah case study. Cities.

[98] Lotfi S., Koohsari M.J. (2009). Measuring objective accessibility to neighborhood facilities in the city (A case study: Zone 6 in Tehran, Iran). Cities.

[99] Bahriny F., Bell S. (2020). Patterns of Urban Park Use and Their Relationship to Factors of Quality: A Case Study of Tehran, Iran. Sustainability.

[100] Addas A. The Creation of Outdoor Spaces and Public Engagement, Jeddah, Saudi Arabia. Proceedings of the Creation/Reaction.

[101] Carmona M. (2019). Principles for public space design, planning to do better. Urban. Des. Int..

